# Easy Read Health Information for People With Intellectual Disabilities: A Systematic Review of the Evidence

**DOI:** 10.1111/jar.70195

**Published:** 2026-02-11

**Authors:** Hollyanna Wilson, Karen Irvine, Regi T. Alexander, Silvana E. Mengoni

**Affiliations:** ^1^ University of Hertfordshire Hatfield UK; ^2^ Hertfordshire Partnership University NHS Foundation Trust Hatfield UK

**Keywords:** accessible information, easy read, health promotion, intellectual disability, patient education, physical health

## Abstract

**Background:**

Easy Read materials are sometimes provided by healthcare services to help people with intellectual disabilities to understand written information. This study examined literature on the development, review, regulation, delivery, and impact of Easy Read health information (ERHI) with the aim of elucidating best practice.

**Methods:**

A systematic review of five bibliographic databases and three grey literature databases was registered, conducted, and synthesised using meta‐aggregation. Studies from 2006 onward regarding ERHI for individuals with intellectual disabilities were included.

**Results:**

The twenty‐nine included studies revealed variability in ERHI development, review, and quality control processes. Individuals with intellectual disabilities valued ERHI and assistance in appraising it, however empirical evidence of ERHI effectiveness was limited.

**Conclusions:**

ERHI's empirical evidence base is underdeveloped and largely consists of low‐quality research. Subjective and inconsistent application of guidance leads to variable ERHI quality. Standardised resources and rigorous research are needed to evaluate ERHI as a health education intervention.

## Lay Summary

- Easy Read health information is made, checked, and used in different ways by different people.
- Some Easy Read health information is too difficult for people with intellectual disabilities to read and understand, even though it says it is ‘Easy Read’.
- Most people with intellectual disabilities like to have support to look at Easy Read Information, and certain strategies can be used to aid understanding.
- Some people with intellectual disabilities find Easy Read health information clear and easy to understand, but more research is needed before we can judge how helpful it is.

## 1 Introduction

Diverse and lifelong speech, language, and communication difficulties are recognised as a core feature of intellectual disability (American Psychiatric Association 2022; Royal College of Speech and Language Therapists 2023). Although no definitive prevalence rate has been established, research indicates that 60%–90% of people with intellectual disabilities have receptive and expressive language difficulties, and up to 95% have reduced literacy levels (Rudd et al. 2003; Royal College of Speech and Language Therapists 2023). Cognitive ability predicts expressive and receptive language proficiency in this population (Smith et al. 2020; Royal College of Speech and Language Therapists 2023), however, language needs are heterogenous (Baker et al. 2010). Challenges typically encompass multiple domains including phonology, vocabulary, and semantics (Abbeduto and Thurman 2022). Grammatical and syntactic difficulties are also prevalent, affecting the construction, comprehension, and contextual application of sentences in oral and written language (Adlof and Catts 2015). Furthermore, literacy difficulties affecting phonemic awareness, decoding, and word recognition often delay or impair functional reading and writing in this population (Alquraini and Rao 2019).

Language skills and cognitive capability are key components of communicative health literacy (Chinn 2016; Geukes et al. 2019); defined as the ability to effectively acquire, comprehend, assess, and apply health related information to promote and sustain wellbeing (Nutbeam 2000). People with intellectual difficulties often exhibit reduced communicative health literacy, reflecting not only cognitive‐linguistic differences, but emotional, social, and societal obstacles (Chinn 2016). Communication and health literacy difficulties have been linked to personal determinants of health, including reduced service engagement, higher‐risk lifestyle factors, and difficulties with self‐management of chronic conditions (Public Health England 2015), which may bring about increased urgent care usage, longer inpatient admissions, higher healthcare costs, poorer health and wellbeing outcomes, and increased mortality (Berkman et al. 2011; Kickbusch et al. 2013; Heslop et al. 2014; Public Health England 2015; Emerson and Baines 2011; Coughlin et al. 2020; NHS England 2023a).

While co‐occurring genetic and neurodevelopmental conditions account for some adverse health and wellbeing outcomes for people with intellectual disabilities, communication difficulties are one of the most frequently cited contributors to pervasive health disparities (Krahn and Fox 2014). Speech, language and communication challenges are often compounded by environmental, social, and systemic factors such as transportation issues, staff shortages, and geographic disparities in resource allocation. Such misalignment between healthcare supply and population demand often results in unmet need (Rodriguez Santana et al. 2023). Addressing and meeting diverse communication needs may therefore improve patient engagement, enhance health literacy, and reduce disparities in health outcomes (NHS England 2018; Public Health England 2020).

Considering the predisposition of people with intellectual disabilities to serious health problems (Liao et al. 2021) and communication difficulties (Royal College of Speech and Language Therapists 2023), enhancing health literacy is an international health priority (World Health Assembly 58 2005; Office of the United Nations High Commissioner for Human Rights 2006). In response to the challenges posed by reduced health literacy, legislation in many contexts mandates “reasonable adjustments” (Equality Act 2010) to ensure accessible information for people with disabilities (Royal College of Speech and Language Therapists 2013; Public Health England 2020; Department of Health and Social Care 2023). The Equality Act (2010) legally requires public sector organisations to make necessary and proportionate changes to remove or reduce disadvantage associated with disability. Furthermore, the Accessible Information Standard (NHS England 2025) stipulates that public health and social care services must ensure equitable access to information for disabled individuals. Although international governance champions the right to receive accessible, reliable, and relevant health information through suitable and supportive means (Office of the United Nations High Commissioner for Human Rights 2006; Office of the United Nations High Commissioner for Human Rights, World Health Organization 2008), evidence of its practical application remains limited (Terras et al. 2021). Governance recognises the multifaceted, intersecting, and dynamic communication support needs of this population. However, resource constraints hinder the implementation of bespoke strategies in the current healthcare climate (Baker et al. 2010; WHO 2025).

One widely implemented reasonable adjustment is the provision of information in what is referred to as an ‘Easy Read’ (ER) format. Easy Read refers to written information which is adapted to reduce complexity and enhance comprehensibility for people with intellectual disabilities. Published guidelines agree on some key components of ER including simplified vocabulary, clear layout, supportive images, and concise content (Lindholm and Vanhatalo 2021). However, there is no current consensus on ER's essential criteria. Guidelines differ in their scope, emphasis, and implementation, resulting in considerable variation in the quality and accessibility of ER materials (González‐Sordé and Matamala 2024). Public services worldwide employ ER as a generic means of meeting the cognitive and communication needs of people with intellectual disabilities (Lindholm and Vanhatalo 2021). In the late 20th century, the concept of ER gained prominence through self‐advocacy groups who championed user‐led content (Lindholm and Vanhatalo 2021). Easy Read emerged not only as a catalyst for social change; reshaping how the needs of people with intellectual disabilities were acknowledged and addressed, but also as a viable commercial practice (Lindholm and Vanhatalo 2021).

ER formats aim to facilitate reading comprehension by adapting information to match the ability of the recipient (Lindholm and Vanhatalo 2021). According to Logogen (Patterson and Shewell 1987) and Dual Route (Coltheart et al. 2001) models, ER may facilitate word recognition through sans‐serif fonts (Hojjati and Muniandy 2013), support lexical retrieval by using high‐frequency words in basic grammatical constructions (Schmutz et al. 2019), and accelerate semantic access through image use (Rivero‐Contreras et al. 2021). Furthermore, Relevance Theory (Sperber and Wilson 1986) suggests that ER formats may reduce the cognitive load of language processing, resulting in increased *relevance* to the reader. Sperber and Wilson posit that the simpler information is to understand, encode, and interpret, the more deserving it is of the receiver's attention. Information deemed *relevant* by the receiver is more effectively attended to and thus more likely to incur “positive cognitive effects” (p. 251) such as knowledge transfer, contextual application, or behaviour change. While ER theoretically aligns with these conceptualisations of information processing, empirical evidence of its positive cognitive effects is scarce (Sutherland and Isherwood 2016; González‐Sordé and Matamala 2024).

Despite the proliferation of Easy Read health information (ERHI) as a reasonable adjustment for people with intellectual disabilities (Tuffrey‐Wijne et al. 2014), Chinn and Homeyard (2017) concluded, through a systematic review, that there was insufficient evidence for its effectiveness. Indeed, there was a dearth of high‐quality evidence demonstrating enhanced comprehensibility, knowledge transfer, or associated behaviour change. Chinn and Homeyard identified empirical challenges including lack of consensus on expected outcomes, suboptimal evaluation measures, and low standards of methodological rigour. Crucially, they recommended that future research employs diverse and rigorous methods to capture real‐world ERHI impact beyond uptake and adherence.

This systematic review, conducted 8 years after the publication of Chinn and Homeyard's seminal study, explored whether contemporary literature has addressed gaps in this field. Our novel research questions aimed to discern the effectiveness of ERHI interventions whilst elucidating current practices. By critically evaluating clinical and research approaches to the development and evaluation of ERHI, we sought to determine whether previously reported shortcomings stem from the intervention itself, or from empirical deficiencies.

## 2 Aims

This study aimed to identify and critically evaluate literature reporting on the development, quality control, delivery, and impact of ERHI for adults with intellectual disabilities.

We addressed the following questions:

1. What guidance is used when developing ERHI?
2. Which quality control processes are used to assess whether ERHI meets proposed standards?
3. Which support strategies are used when delivering ERHI and are these effective?
4. What evidence supports Easy Read as an effective way of presenting physical health information to people with intellectual disabilities?

## 3 Methods

### 3.1 Reporting Standards

This systematic review was reported in line with the 2020 Preferred Reporting Items for Systematic Reviews and Meta‐Analyses (PRISMA) updated guideline (Page et al. 2021) and the Joanna Briggs Institute (JBI) manual for evidence synthesis (Aromataris et al. 2024). The quality of reporting was assessed using the PRISMA 2009 checklist (Moher et al. 2009) (Table S4). The review protocol was registered with PROSPERO (CRD42024546386).

### 3.2 Searching

A systematic search was made office electronic bibliographic databases in May 2024, and again in October 2024. The databases searched were: United States National Library of Medicine's bibliographic database (MEDLINE), National Library of Medicine (PubMed), American Psychological Association (PsycNet), Excerpta Medica Database (Embase) and Cumulative Index to Nursing & Allied Health Literature (CINAHL). Further scholarly references were identified via forward and backward searching of the literature by hand and using ‘ResearchRabbit’ (Research Rabbit n.d.). The Health Education England Knowledge and Library Services ‘Grey Literature’ resource (Health Education England 2023) was used to identify three appropriate grey literature databases: Google Scholar, the Bielefeld Academic Search Engine (BASE), and Open Access Theses and Dissertations (OATD). Search terms (Table S1) were based on the PICO framework (Richardson et al. 1995) and informed by a review of analogous literature and consultation with clinicians and researchers working in the field of intellectual disability. Our review included publications from January 2006 onwards using a custom date extraction category of January 2006–October 2024. This date threshold was determined to coincide with the publication of the Convention on the Rights of Persons with Disabilities (Office of the United Nations High Commissioner for Human Rights 2006); the first international governance to set out accessibility requirements for health information.

### 3.3 Study Selection

Studies were screened according to the eligibility criteria in Table 1. Eligible studies were retrieved and imported into the Rayyan screening and selection web tool (Ouzzani et al. 2016). After duplicate removal, the first and second authors independently screened titles and abstracts against inclusion criteria (see Table 1), then retrieved and assessed the full texts of all remaining studies for eligibility.

**TABLE 1:** Inclusion and exclusion criteria.

Parameter	Included	Excluded
Publication type	Published in or translated into English. Original research. Published in full. Published in a peer‐reviewed journal.	Not available in English. Editorials, commentaries, or opinion pieces. Abstracts, posters, or presentations. Magazine articles, blogs, websites.
Study design	Qualitative, quantitative and mixed methods design.	Systematic reviews, narrative reviews, protocols.
Participants	Adults (≥ 18 years) with intellectual disability. Adults (≥ 18 years) with a personal and/or professional connection to the intellectual disability community, including but not limited to: family members, carers, health and social care professionals, governing bodies, or commissioners/producers/publishers of Easy Read information.	Children and young people (< 18 years). Adults with: – Learning difficulties, for example, dyslexia – Acquired or progressive cognitive impairment attributable to injury/disease after the age of 18 years – Visual and/or hearing impairment, in the absence of congenital cognitive impairment.
Intervention	Easy Read (or synonymously termed) health‐related information in written, printed and/or digital formats.	Accessible information only in multimedia formats, for example, video/audio. Accessible information tailored to non‐intellectual disability cohorts, for example, plain language, clear print. Easy Read material not on the topic of physical health.
Comparison	Relevant comparators (e.g., written health information used in standard care) or no comparators.	Irrelevant comparators, for example, alternative health education intervention (videos, groups etc.)
Outcome	Reports qualitative or quantitative data pertaining to research questions, that is: Easy Read guidance ERHI quality control practices ERHI delivery support strategies ERHI Effectiveness	No empirical data reported. Data reported does not align with research questions.
Setting	Any setting (so long as other inclusion criteria are met).	

### 3.4 Data Extraction and Synthesis

Key data from included studies were extracted, recorded, and grouped using a summary table (Table S2) based on that of (Chinn and Homeyard 2017) comprising: year of publication, aims, participants, design and measures, and findings. Findings were organised by research question and presented in four sub‐columns. Evidence was synthesised using a convergent integrated approach (Aromataris et al. 2024). Data from qualitative, quantitative, and mixed‐methods studies were transformed into textual descriptions. Findings were reported using meta‐aggregation, a method that facilitates the categorisation of data based on thematic similarity. This approach was selected for its ability to preserve original interpretations (Lockwood et al. 2015) while integrating findings to form prevalence‐based recommendations for policy and practice (Aromataris et al. 2024).

### 3.5 Quality Assessment

We employed the Quality Assessment with Diverse Studies (QuADS) Tool (Harrison et al. 2021) to minimise researcher reporting bias. The QuADS tool demonstrates face and content validity, making it suitable for use in systematic reviews involving mixed or multi‐methods health research (Harrison et al. 2021). Studies were evaluated by the first author using the QuADS criteria, with 10% independently reviewed by the second author to establish inter‐rater reliability. Studies were assigned a score ranging from zero to three for each of the 13 criteria. The cumulative score was then converted into a percentage, facilitating comparison of overall quality across included studies.

## 4 Results

**FIGURE 1:**
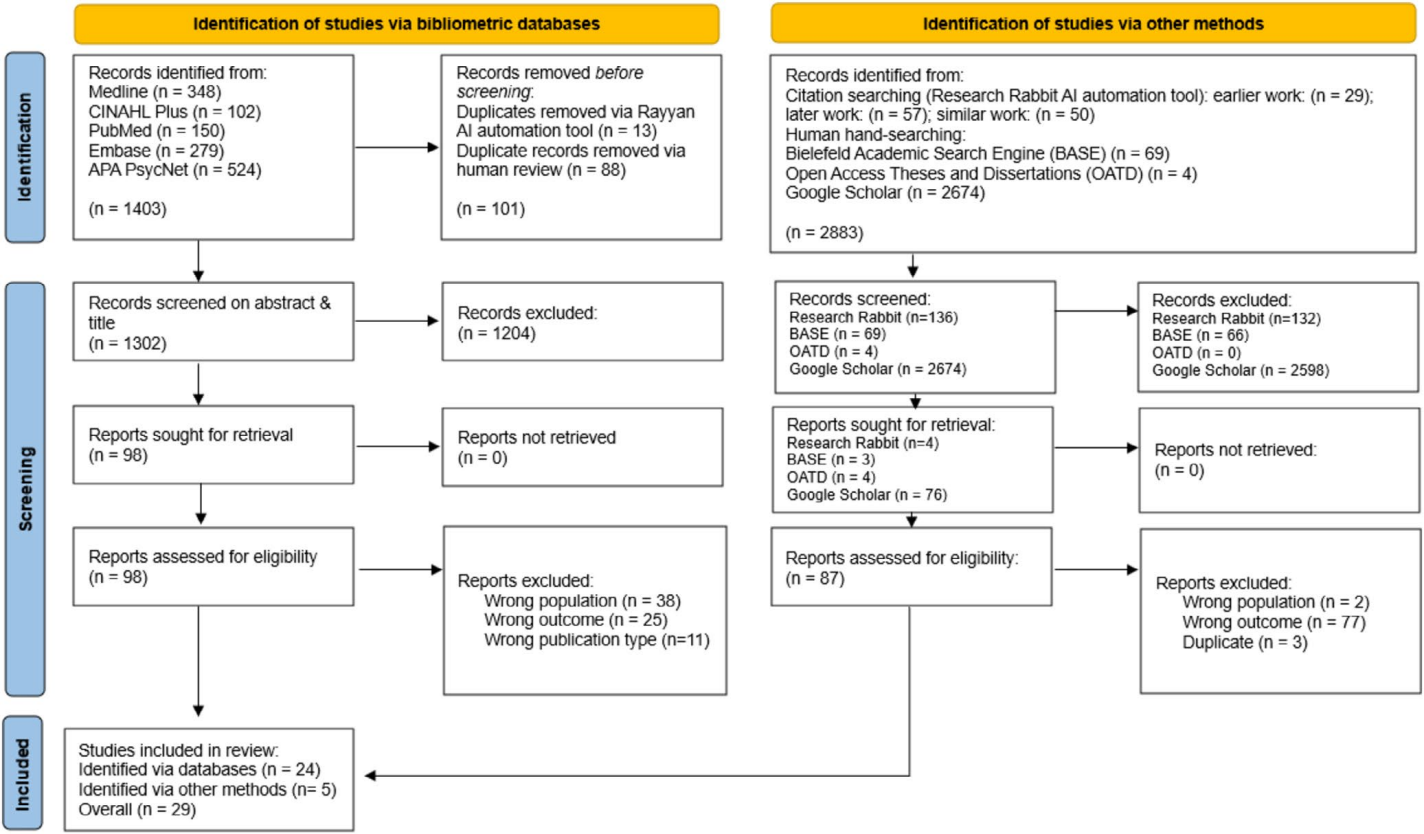
PRISMA 2020 flow diagram for new systematic reviews.

Search outcomes are documented using a PRISMA flow diagram (Page et al. 2021) (see Figure 1). Searches in May 2024 yielded 4026 results, 27 of which met inclusion criteria. The updated search in October 2024 yielded a further 260 results, of which 257 were excluded after title and abstract screening by the first author. Full‐text review of the remaining three articles led to the exclusion of one based on Table 1 criteria, leaving two additional studies that met inclusion criteria.

Overall, 29 publications were eligible for inclusion (Table S2). Of the 29 publications, 23 reported information pertinent to question 1 (guidance), 24 to question 2 (quality control), 19 to question 3 (communication support), and 14 to question 4 (Effectiveness) (Table S2). Twenty‐six articles described research conducted in the UK: 21 in England, three in Scotland, one in Wales, and one in Northern Ireland. Two studies were conducted in Austria, and one in Australia. Numbers of professionals and caregivers were predominantly unspecified or estimated, but numbers of people with intellectual disabilities were more clearly documented. The number of people with intellectual disabilities involved in the included studies ranged from two to 60, though most studies included 20 or fewer people with intellectual disabilities (median = 7).

### 4.1 Quality Appraisal of Completed Studies

QuADS scores (Table S2, column 2) demonstrated considerable variation in the methodological and reporting quality of the included publications (see Table 2). The median score was 20 (51%), with a range of five to 38 out of a possible 39; where higher scores represent superior quality. Of the 29 publications, 19 referenced relevant theoretical or conceptual frameworks, 17 stated clear aims, and 17 described their setting and target population. Twenty‐two studies relied on convenience sampling and lacked baseline measures or control groups. While all studies used appropriate designs, 17 could have benefited from the application of additional methods to offer a stronger analysis. Data collection tools and processes were transparently reported in 11 publications, aligned with research aims in nine, and were effectively justified in just six. While 24 papers employed suitable analytic methods, only seven provided comprehensive rationale for these. Finally, only five papers provided a comprehensive discussion of strengths and limitations.

**TABLE 2:** Overall QuADS scores for each parameter.

QuADS parameter	Overall score (%)
Theoretical or conceptual underpinning to the research	84
Statement of research aim/s	70
Clear description of research setting and target population	76
The study design is appropriate to address the stated research aim/s	78
Appropriate sampling to address the research aim/s	55
Rationale for choice of data collection tool/s	38
The format and content of data collection tool is appropriate to address the stated research aim/s	33
Description of data collection procedure	64
Recruitment data provided	47
Justification for analytic method selected	40
The method of analysis was appropriate to answer the research aim/s	66
Evidence that the research stakeholders have been considered in research design or conduct	22
Strengths and limitations critically discussed	43

### 4.2 Guidance on Easy Read Information

In this section research question 1 is addressed: *which guidance is used when developing ERHI?*

Of the 29 papers included in this systematic review, 21 described the development process of ERHI. Publications variously comprised nine service improvement projects, three pilot studies, one service evaluation, one feasibility study, one randomised controlled trial, and six qualitative studies employing questionnaires (*n* = 2), focus groups (*n* = 2), interviews (*n* = 1) and content analysis (*n* = 1) (Table S2; Q1). In nine of these publications, ER guidance was not referred to where this would have been applicable. The remaining 12 studies explicitly referred to guidance for the creation of ER information but offered little elaboration surrounding the review or application of guidance.

Across the included studies, 20 distinct ER guidance documents were mentioned (see Table 3). In five publications detailing resource development or practitioner experiences, two or more different guidance documents were referred to (King 2011; Howieson and Clarke 2013; Chinn 2020; Doherty et al. 2021; Dam et al. 2023). Sources of guidance included charities, non‐profit organisations, international consortiums, the UK Government, the National Health Service (NHS), researchers (individuals and centres), a private company, and an individual clinician. Guidelines authored by Mencap, Change, and the UK Department of Health were most cited, appearing in two publications each.

**TABLE 3:** Sources of Easy Read guidance cited in the included papers.

Author	Title	Study/studies citing guidance
Change (2015)	How to make information accessible	Chinn (2019a) Waight and Oldreive (2020)
Department of Health and Social Care (2010)	Making written information easier to understand for people with learning disabilities	Chinn (2019a) Waight and Oldreive (2020)
Mencap (2000)	Am I making myself clear?	King (2011) Doherty et al. (2021)
Department of Health (2003)	Toolkit for producing patient information	King (2011) Howieson and Clarke (2013)
Inclusion Europe (n.d., first published 2014/15)	Information for all: European standards for making information easy to read and understand	Dam et al. (2023)
Accessible Information Working Group (2011)	Make it Easy: A guide to preparing easy to read information	Toussi and Cithambaram (2022)
Belfast Health and Social Care Trust (2015)	Making communication accessible for all: A guide for health and social care (HSC) staff	Waight and Oldreive (2020)
Freyhoff (1998)	Make it simple: European guidelines for the production of easy‐to‐read information for people with learning disability for authors, editors, information providers, translators and other interested persons	Yaneva et al. (2016)
International Federation of Library Associations and Institutions (2010)	Guidelines for easy‐to‐read material	Waight and Oldreive (2020)
“Lead Intellectual Disability Nurse” (n.d.)	Unknown	King (2011)
Mencap (2008)	Make it clear	Howieson and Clarke (2013)
Marsay (2017)	Accessible information standard	Doherty et al. (2021)
NHS England (2018)	Guide to making information accessible for people with a learning disability	Toussi and Cithambaram (2022)
NHS Scotland Learning Disabilities Managed Care Network (2007)	Guidance for making written information easier to understand	Dawson (2011)
Norah Fry Research Centre and Royal National Institute of the Blind (2004)	Information for all	Waight and Oldreive (2020)
Office for Disability Issues and Department for Work and Pensions (2018)	Government advice on inclusive communication	Doherty et al. (2021)
The Clear Communication People (2009)	A guide to producing easyread information	Waight and Oldreive (2020)
“UK government”	Unknown guidelines	Mander (2015)
Unknown	Web content accessibility guidelines	Dam et al. (2023)
Unknown	“Easy to Read” “Leicht Lesen” guidelines	Dam et al. (2023)

### 4.3 Quality of ERHI

In this section, research question 2 is addressed: *which quality control processes are used to assess whether ERHI meets proposed standards?*

In this paper, ‘quality control’ refers to the processes used to test ERHI materials against imposed standards, using either objective measures (e.g., readability metrics) or subjective measures (e.g., personal judgements of accessibility)‘Quality’ denotes the *accessibility* and *acceptability* of ERHI resources, as evidenced by quality control processes.

Twenty‐four descriptive, qualitative, and experimental studies made reference to ERHI quality control processes within research and clinical practice. Seventeen of these reported quality judgements in various forms, ranging from the opinions of single clinicians to numerical values calculated using established readability formulae (Table S2; Q2).

The 24 relevant studies employ a range of methodologies to address their diverse research aims. Some authors examine the acceptability, readability, and effectiveness of ERHI using mixed methods (*n* = 6) or quantitative, experimental designs (*n* = 5), while others explore the experiences of service users, healthcare staff, or resource creators using descriptive (*n* = 5) or qualitative methods such as interviews and focus groups (*n* = 8). Twenty‐three of the 24 relevant studies employed similarly structured, yet non‐standardised sequences. Quality control processes typically began with individual or group ERHI development, continued with iterative review and revision employing objective or subjective, and ended with formal approval by their respective development and/or review panel.

Differences in study aims were reflected in the scope and rigour of quality control processes applied. Development‐focussed studies typically employed robust, thorough, and user‐centred quality control through iterative stakeholder review. Similarly, studies with evaluative or analytical aims applied validated formal testing methods such as readability formulae with embedded linguistic analysis. In contrast, implementation‐focussed studies relied on more pragmatic approaches, such as informal feedback, with limited reporting of explicit quality judgements. Studies engaging diverse stakeholders, including people with intellectual disabilities, often reported greater confidence in ERHI quality, albeit with extended creative timelines. Across 23 of the 24 studies, ERHI underwent extensive scrutiny by up to 21 collaborators who reviewed up to six drafts (Kotwal et al. 2020) prior to approval. Contrastingly, one service evaluation comprising a poorly documented review panel (King 2011) reported unanimous endorsement without revisions despite the absence of explicit quality judgements.

Stakeholder collaboratives were typically diverse, and studies did not provide justification for their composition. Across the studies, 11 clinical disciplines and 15 non‐clinical partners were represented in configurations aligned with each study's aims, objectives, and methods (Table S3). Clinicians comprised doctors, nurses, allied healthcare professionals, midwives, psychologists, pharmacists, and social workers. Non‐clinical parties included corporate and managerial healthcare staff, academics, external service providers, carers, advocates, and people with intellectual disabilities.

Thirteen studies included individuals with intellectual disabilities in their development or review collaboratives. However, just two service evaluations and one mixed‐methods study described coproduction activities from ERHI conception to completion (Dawson 2011; Sawhney et al. 2017; Cox et al. 2021). Similarly, clinicians with expertise in health and communication were involved inconsistently in content creation. Nurses and doctors were incorporated within creative collaboratives in just under half of the relevant publications, and speech and language therapists were included in less than a quarter. Furthermore, medical, nursing, and allied health professionals only served as ERHI reviewers in nine studies where they possessed specialist expertise in the field of publication (e.g., midwives for birthing‐related topics). Instead, managerial and corporate stakeholders were favoured as end‐stage reviewers of ERHI outputs.

Of the 17 publications that described the quality of ERHI resources, 11 presented subjective quality judgements and six reported objective quality scores (Table S2, Q3). Where quality was subjectively defined, it was inferred through review panel sign‐off (King 2011; House et al. 2018; Kotwal et al. 2020; Cox et al. 2021; Toussi and Cithambaram 2022), national interest in ERHI materials (Dawson 2011; Kelly 2011; Howieson and Clarke 2013; Denyer 2016), and positive feedback from healthcare professionals (Dawson 2011; Howieson and Clarke 2013) and people with intellectual disabilities (Porter et al. 2012; House et al. 2018). Six studies documented more specific feedback regarding the clarity (Porter et al. 2012; Cox et al. 2021), readability (House et al. 2018; Kotwal et al. 2020), layout (Cox et al. 2021), and usability (Kotwal et al. 2020) of ERHI resources. Though ERHI was generally deemed acceptable by its users, three qualitative studies reported disengagement with ERHI when presented (Mander 2016; House et al. 2018; Bruun et al. 2024); with some people with intellectual disabilities suggesting it belonged ‘in the bin’ (Bruun et al. 2024, p4).

Objective quality standards were explicitly defined in six experimental studies, five of which assessed accessibility, but not acceptability. (Toussi and Cithambaram 2022) were the only authors to develop a custom assessment tool, which evaluated linguistic and extralinguistic factors based on their alignment with ER guidance. (Yaneva et al. 2016; Buell et al. 2020) employed long‐established Flesch–Kincaid Grade Level Readability Formula (Flesch 1948) to discern required education level based on word and sentence length. (Yaneva et al. 2016) judged quality according to an imposed threshold for inclusion (score of≥ 65, reading age of 13–14 years), leading to 5% of purportedly ‘Easy Read’ resources being excluded pre‐experimentally. (Buell et al. 2020) benchmarked their ERHI against resources already in circulation and found that the mean reading age of existing ERHI was 13–14 years; far beyond the reading age of 8 years suggested by contemporary research (Nilsson et al. 2024). (Buell et al. 2024) used automated the coh‐Metrix software (Graesser et al. 2004), which combines cohesion, linguistic, and readability indices, and similarly found a mean reading age of 13–14 years; with one sample equivalent to undergraduate reading level. (Rowlands 2015), using the SMOG index (McLaughlin 1969), also reported undergraduate‐level readability scores across two ERHI samples. (Kotwal et al. 2020), who combined automated computer software integrating multiple readability algorithms (Readable 2015) with subjective evaluation by review collaboratives, were the only researchers to describe a resource with a reading age of 8 years.

Both qualitative and quantitative evidence indicated that many ERHI materials employed in clinical and research contexts were not accessible. Indeed, eight studies described the acceptance and utilisation of ‘*more‐or‐less easy‐read*’ resources (House et al. 2018, 4) which did not objectively or subjectively adhere to quality standards (Mander 2015; Rowlands 2015; Yaneva et al. 2016; Kotwal et al. 2020; House et al. 2018; Cox et al. 2021; Dam et al. 2023; Douglass et al. 2023). In 16 publications, ERHI was deemed unfit for purpose from an accessibility perspective due to non‐compliance with Easy Read guidelines (Rowlands 2015; Toussi and Cithambaram 2022; Dam et al. 2023), inaccuracies (Kotwal et al. 2020), incompleteness (Chinn 2019a; Douglass et al. 2023), biased and compliance‐focussed messages (Chinn 2017, 2019a), suboptimal presentation (Douglass et al. 2023), or linguistic complexity, that is, high equivalent reading age (Rowlands 2015; Yaneva et al. 2016; Buell et al. 2020, 2024; Kotwal et al. 2020; Cox et al. 2021).

### 4.4 Communicative Support

In this section research question 3 is addressed: *which support strategies are used when delivering ERHI and are these effective?*

Eighteen of the included studies examined communicative support in conjunction with the provision of ERHI (Table S2; Q3). Ten publications describe communication support strategies used in clinical practice or research, five of which report observations of such support (Chapman 2014; Rowlands 2015; Mander 2016; Chinn 2019b, Waight and Oldreive 2020), and five provide descriptive reflections by clinicians or researchers (Porter et al. 2012; Marriott et al. 2015; Denyer 2016; Heslop et al. 2019; Buell et al. 2020). Findings from 16 studies suggested that the involvement of a support person was perceived, by people with intellectual disabilities, to be a beneficial and highly valued element of the interaction. Furthermore, in three studies (Chapman 2014; Rowlands 2015; Chinn 2020) individuals with intellectual disabilities highlighted that while they thought ERHI was beneficial, they felt the resource alone was insufficient without verbal facilitation, the resource alone was insufficient without verbal facilitation.

The competencies required to fulfil the role of the support person were underexplored in this body of literature, however some personal attributes were proposed. Intuition (Dam et al. 2022), unconditional positive regard (Mander 2015), empathy, patience, and respect for autonomy, privacy, and dignity (Mander 2015, 2016) were deemed essential to the role, yet communication skills were not acknowledged as a core capability. In Mander's paper (Mander 2015), the role is described as specialist and requiring skills beyond those of typical health and social care practitioners, however no defined role requirements or qualification criteria were suggested.

Despite limited clarity on role specifications, 15 studies detailed communication scaffolds used by support persons. These included assisting with the physical handling or positioning of materials (Chinn 2020), introducing topics to prompt discussion (Chinn 2020), and working to establish and maintain joint attention (Mander 2016; Chinn 2019b; House et al. 2018). The primary role of the support person was to supplement written content (Porter et al. 2012; Chapman 2014; Marriott et al. 2015; Sawhney et al. 2017; Heslop et al. 2019; Chinn 2020) through clarification and elaboration. Clarification involved defining abstract terms (Rowlands 2015; Mander 2016; Buell et al. 2020; Waight and Oldreive 2020), simplifying language (Porter et al. 2012), offering supplementary images (Marriott et al. 2015), highlighting key words (Chapman 2014), or using communication aids such as Makaton (Howieson and Clarke 2013) and sign language (Heslop et al. 2019). Elaboration comprised bespoke scaffolding through the provision of concrete examples (Chapman 2014; Mander 2016) and practical demonstrations (Chapman 2014; Marriott et al. 2015; Cox et al. 2021). Encoding of health information was supported through verbal summarising (Buell et al. 2020), repetition (Waight and Oldreive 2020), discussion (Mander 2016; Wilson et al. 2018; Cox et al. 2021) and encouraging questions to promote deeper understanding (Porter et al. 2012).

In spite of widespread praise of support people, three studies called into question the appropriacy and benefit of this arrangement. Chinn (2019b) identified potential drawbacks, including biased messaging and the provision of unsolicited advice due to differing experiences and perspectives. Buell et al.'s randomised controlled trial (2020) found that support person involvement did not have a statistically significant impact on knowledge transfer, while Rowlands single‐case within‐participants study (2015) found that verbal mediation had a negative impact on post‐intervention comprehension scores in 17% of cases.

### 4.5 Evidence for the Benefit of Easy Read Health Information

In this section, research question 4 is addressed: *what evidence supports Easy Read as an effective way of presenting physical health information to people with intellectual disabilities?*

Fourteen studies offered evidence, to varying degrees of robustness, for the benefit of ERHI (Table S2; Q4). Eleven studies described qualitative research methods including recorded observations (Chapman 2014; Mander 2016), interviews (Porter et al. 2012; Chapman 2014; Mander 2015; Chinn 2020; Dam et al. 2023), focus groups (Howieson and Clarke 2013; Chapman 2014; Mander 2015; Waight and Oldreive 2020; Dam et al. 2023), questionnaires (House et al. 2018; Dam et al. 2022) and narrative accounts (Kelly 2011; Marriott et al. 2015; Heslop et al. 2019). Four studies employed analytical experimental designs: (Rowlands 2015) single case within participant design, (Wilson et al.'s 2018) participatory randomised parallel study, (Buell et al.'s 2020) randomised controlled trial, and (Dam et al.'s 2022) pilot study.

Qualitative evidence from ten studies took the form of feedback from healthcare professionals and/or people with intellectual disabilities. Subjective responses included reports of improved understanding (Kelly 2011; Porter et al. 2012; Wilson et al. 2018), decision making (Porter et al. 2012), self‐direction (Porter et al. 2012; Wilson et al. 2018), confidence (Porter et al. 2012; Mander 2015; Wilson et al. 2018; Heslop et al. 2019), participation (Chapman 2014; Wilson et al. 2018) and healthcare satisfaction (Chapman 2014). Publications that explored associated behaviour change commented on anecdotal increases in appointment attendance and intervention uptake (Marriott et al. 2015; Heslop et al. 2019), but no studies reported any change in objective measures of physical health.

Findings from the four experimental studies assessing knowledge transfer were varied. Rowlands' single case within‐participant study (Rowlands 2015) found that while independent use of ERHI increased correct responses, comprehension scores were higher with communicative support. (Dam et al. 2022) reported 100% response accuracy with assisted ERHI use in their pilot study, but noted that the lack of baseline assessment may have skewed data. Wilson et al.'s participatory randomised parallel study (2018), which employed baseline measures, reported statistically significant improvements in knowledge and skills scores associated with ERHI. Knowledge scores were maintained for a further six months, however the presence of support people both during and post‐intervention introduced a major uncontrolled variable. Finally, Buell et al.'s randomised controlled trial (Buell et al. 2020) found no significant improvement in comprehension, retention, or knowledge application with or without mediation, instead identifying intrinsic language ability as a stronger predictor of performance.

Overall, while evidence suggests potential benefits to individuals with intellectual disabilities, limited methodological rigour and reporting quality prevent definitive conclusions regarding ERHI's efficacy. Although all included publications reported some degree of positive impact, none provided evidence of statistically significant knowledge transfer or behaviour change directly attributable to ERHI.

## 5 Discussion

Despite ERHI's widespread application in clinical practice over the last two decades, its empirical evidence base remains limited. This review identifies several challenges encountered by researchers and clinicians in the development, use, and evaluation of ERHI. In the included publications, guidance for developing ERHI was drawn upon and applied inconsistently, with key stakeholders often excluded from the creative process. Quality assessment methods presented limitations, resulting in the dissemination of suboptimal ERHI resources. ERHI was delivered by support people with varying skills and experience, who offered differing levels of assistance. Notwithstanding its perceived value, robust evidence supporting the effectiveness of ERHI for people with intellectual disabilities was scarce.

Where ER guidance was utilised, multiple sources were drawn upon. Although extensive guidance exists, its lack of formal accreditation or enforcement likely contributes to inconsistent implementation. ER guidance is often marked by ambiguity, inconsistency, and limited empirical and theoretical grounding (González‐Sordé and Matamala 2024). Indeed, it frequently overlooks the extralinguistic and biopsychosocial factors central to language processing which should underpin all approaches to accessible information (Laverack 2017). It is, however, crucial to recognise that rigid guidance may be limiting. While consensus on core Easy Read characteristics is likely to support consistent practice, associated frameworks must afford flexibility in order to meet individual needs.

Where quality control processes were documented, these were predominantly subjective. Quality judgements were presented according to the views and opinions of clinical and non‐clinical professionals, the recruitment and selection of whom often lacked justification (Bernabé and Cavallo 2022). People with intellectual disabilities were not consistently involved in quality control processes despite their critical status as the ERHI end‐user, possibly reflective of their broader societal marginalisation (Bishop et al. 2024). While there is no empirical evidence linking coproduction to improved ERHI quality, it remains a morally and ethically imperative practice due to its emphasis on inclusivity, empowerment, and respect for the voices of those it serves (UK Government: Cabinet Office and Disability Unit 2021; NHS England 2023b).

Objective quality control methods also presented fundamental limitations. Readability formulae lack sensitivity, were developed for neurotypical populations, and often exclude linguistic, contextual, and practical factors crucial to accessibility (Fajardo et al. 2013; Buell et al. 2024). Language processing theories (Patterson and Shewell 1987; Coltheart et al. 2001) underscore the importance of format, design, and imagery; elements that are often unaccounted for when readability formulae are employed in isolation. Given the limitations of subjective and objective appraisal methods when applied independently, combining these may enable more comprehensive quality control.

Even with quality control mechanisms in place, ERHI in the included studies was not always deemed accessible or acceptable to people with intellectual disabilities. Many resources promoted as “Easy Read” failed to meet suggested objective and subjective quality standards due to reported inaccuracies, incompleteness, biased messaging, poor presentation, and complex language. Regardless of quality, subjective reports of improved health‐related knowledge and behaviour change relating to its provision were abundant. People with intellectual disabilities described informative, confidence‐building, and empowering ERHI as a contributor to increased understanding and improved health outcomes. Healthcare professionals mirrored this sentiment, reporting observations of enhanced understanding, decision making, and autonomy. It remains difficult, however, to separate the impact of the resource from that of the support person, who some people with intellectual disabilities perceived to be more important than the resource itself (Chinn 2019a).

The support person's involvement was clearly valued by individuals with intellectual disabilities when accessing ERHI, and professionals considered this role to require a specialist, yet undefined skillset (Newman et al. 2023). Publications positioned the support person as central to ERHI use through their provision of clarification and elaboration, though the appropriacy of this hierarchical dynamic was queried (Mander 2016). Overall, despite praise for their responsive, flexible, and individualised assistance, support person involvement had limited measurable impact on knowledge transfer in experimental studies (Rowlands 2015; Wilson et al. 2018; Buell et al. 2020; Dam et al. 2022).

While ERHI is internationally purported as good practice (Lindholm and Vanhatalo 2021), the research available does not reliably demonstrate effectiveness. This body of literature presented little experimental evidence that ERHI, with or without support, directly facilitated knowledge transfer, informed decision making, or affected the health behaviours and outcomes of people with intellectual disabilities. This review synthesises a broad range of evidence, both endorsing and contesting the impact of ERHI. However, the vast heterogeneity in the methodological and reporting quality of included publications highlights the need for more rigorous research to advance understanding within this field.

### 5.1 Strengths and Limitations

This study employed a structured approach to the identification and evaluation of ERHI research, thereby enhancing the reliability of conclusions. The rigorous protocols adopted for searching, selection, and quality assessment reduced risk of author bias, selective reporting, and non‐replicability. Employing a mixed methods systematic review enabled a comprehensive synthesis of the multifaceted evidence in this field, enhancing the clinical applicability of findings (Stern et al. 2020). However, while reviews of this kind provide a broader foundation than single‐method reviews, their heterogenous nature complicates between‐study comparison (Lizarondo et al. 2022). The included studies varied in their aims and research questions, employed designs ranging from observational studies to randomised experiments, and synthesised and articulated evidence using diverse approaches. Consequently, the lack of conceptual and methodological convergence constrained comparison across the literature.

In addition, differences in quality and reporting standards across the qualitative and quantitative studies may have introduced bias, affecting the overall rigour of the review. While the QuADS was employed to assess the quality of studies across diverse methodologies, we acknowledge that it is more applicable to quantitative methodologies and also lacks cut‐off thresholds to distinguish between high‐ and low‐quality studies. Nevertheless, the QuADS facilitated pragmatic identification of limited high‐quality evidence, with 48% of included studies scoring below 50% on its appraisal criteria. Consequently, the strength of evidence supporting or refuting ERHI effectiveness is limited by the low quality of the literature reviewed.

Finally, we recognise that the lack of consultation with experts by experience is a major shortcoming, particularly in light of critiques of the reviewed literature. Evidence highlights the value of involvement of patient and public engagement throughout the systematic review process (Zhou et al. 2024), which should be intentionally sought to improve methodological and reporting practices.

### 5.2 Implications

**FIGURE 2:**
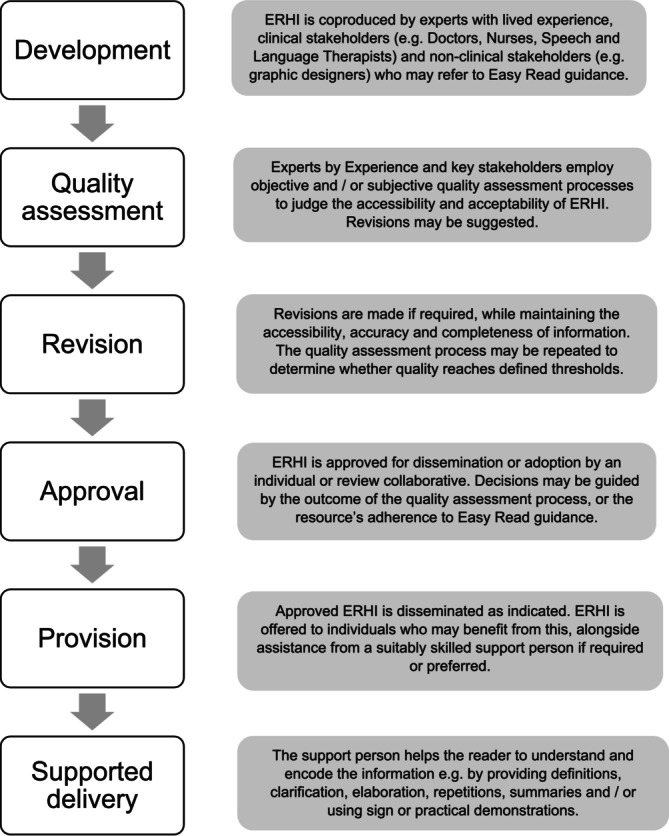
Common practices for the development and delivery of Easy Read health information (ERHI).

Despite its limited evidence base, ERHI's global uptake and endorsement by people with intellectual disabilities suggest it will remain an enduring practice. To address the significant variability in current approaches, research underscores the need for more consistency in ERHI development, quality control, and delivery. The findings of this review highlighted some common processes used by researchers and clinicians when developing ERHI, which have been synthesised to provide an accessible summary of current practice (see Figure 2).

## 6 Conclusion

Similarly to Chinn and Homeyard (2017), we found limited empirical support for ERHI as a health education intervention. Key barriers to reliable evaluations are the lack of consensus on ER guidance and the voluntary nature of its application. In the absence of standardised criteria, quality assessment tools, or accreditation processes, ERHI outputs will continue to vary in quality. Heterogeneity in resource quality hinders meaningful evaluation and, more importantly, exacerbates inequalities in the provision of health information. Future research should therefore begin by empirically validating ER criteria, with embedded coproduction to authentically reflect the views of people with intellectual disabilities. Once core principles are established and agreed upon, tools can be developed to benchmark ERHI outputs and rigorous evaluations can be undertaken.

Given the diversity of people with intellectual disabilities and the range of practitioners developing and delivering ER materials, careful attention to linguistic complexity and communication support is critical to ensure equitable access. Integrating accessibility principles and supportive scaffolds within ER creation, quality control, and provision should result in adaptations that are both theoretically robust and practically effective. SLTs can play a central guiding role in advocating for reasonable adjustments and providing expertise in relation to language simplification, comprehension, and encoding strategies. Crucially, people with intellectual disabilities are best positioned to advise on their own language and communication needs and preferences, underscoring the need for their active and meaningful inclusion in ER research.

## Funding

Hollyanna Wilson is supported by a Ph.D. studentship from the Centre for Health Services and Clinical Research at the University of Hertfordshire.

## Conflicts of Interest

The authors declare no conflicts of interest.

## Supporting information


**Table S1:** Search terms.

**Table S2:** Included publications.

**Table S3:** Stakeholders involved in the Easy Read Health Information development and review process.

**Table S4:** PRISMA 2009 checklist.

## Data Availability

Data sharing is not applicable to this article as no datasets were generated or analysed during the current study.
