# Supplementary material for: Easy Read Health Information for People With Intellectual Disabilities: A Systematic Review of the Evidence
Source: J Appl Res Intellect Disabil. 2026 Feb 11;39(1):e70195. doi: 10.1111/jar.70195 (PMC12893875; doi:10.1111/jar.70195)
Supplement: Supplementary file 1 — Table S1: Search terms. [file JAR-39-e70195-s002.pdf]

Supplementary file 1. *Search terms.*

**Population**	Adult*		
	**AND**		
	"Learning disab*"	**OR**	1) "Intellectual disab*"<br>2) "Developmental disab*"<br>3) "Intellectual developmental disab*"<br>4) "Mental retard*"<br>5) "Mental handicap*"<br>6) "Mental impair*"<br>7) "Mental subnormal*"<br>8) "Pervasive developmental disab*"<br>9) PDD<br>10) "Developmental delay*"<br>11) "Learning difficult*"<br>12) Autis*<br>13) Asperger*<br>14) ASD<br><br>*(Melvin et al., 2022)*
	**AND**		
**Intervention**	"Easy read"	**OR**	1) Easy-read<br>2) "Easy to read"<br>3) Easy-to-read<br>4) "Easier to read"<br>5) "Easy to understand"<br>6) "Easier to understand"<br>7) "EZ read"<br>8) "Easy information"<br>9) "Accessible information"<br>10) "Plain language"<br>11) "Easy language"<br>12) "Easy English"<br>13) "Clear* language"<br>14) "Simp* language"
	**AND**		
**Outcomes**	Health	**OR**	1) "Health condition*"<br>2) "Health problem*"<br>3) "Physical disease*"<br>4) Physical disease exp as MeSH term (including exp "bacterial infections and mycoses"/ or exp virus diseases/ or exp parasitic diseases/ or exp neoplasms/ or exp musculoskeletal disease/ or exp digestive system diseases/ or exp stomatognathic disease/ or exp respiratory tract diseases/ or exp otorhinolaryngologic diseases/ or exp

nervous system diseases/ or exp eye diseases/ or exp male urogenital diseases/ or exp “female urogenital diseases and pregnancy complications”/ or exp cardiovascular diseases/ or exp “hemic and lymphatic diseases”/ or exp “congenital, hereditary, and neonatal diseases and abnormalities”/ or exp “skin and connective tissue diseases”/ or exp “nutritional and metabolic diseases”/ or exp endocrine system diseases/ or exp immune system diseases/ or exp “disorders of environmental origin”/ exp disease)

5) “Physical disorder*”
6) Physical disorder exp as MeSH term (including exp “blood and lymphatic disorders”/exp cachexia/exp cardiovascular disorders/exp chronically ill children/exp digestive system disorders/exp endocrine disorders/exp genetic disorders/exp health impairments/exp immunologic disorders/exp infectious disorders/exp metabolism disorders/exp musculoskeletal disorders/exp neonatal disorders/exp neoplasms/exp nervous system disorders/exp nutritional deficiencies/exp respiratory tract disorders/exp sense organ disorders/exp sensory system disorders/exp skin disorders/exp toxic disorders/exp urogenital disorders/exp vision disorders)
7) “Physical condition*”
8) Comorbidity (Mesh term) and keyword (comorbid*)
9) Comorbid*

(Liao et al., 2021)
