# Supplementary material for: Easy Read Health Information for People With Intellectual Disabilities: A Systematic Review of the Evidence
Source: J Appl Res Intellect Disabil. 2026 Feb 11;39(1):e70195. doi: 10.1111/jar.70195 (PMC12893875; doi:10.1111/jar.70195)
Supplement: Supplementary file 2 — Table S2: Included publications. [file JAR-39-e70195-s001.docx]

| **Supplementary file 2:** *Included publications.* | | | | | | | | | |
| --- | --- | --- | --- | --- | --- | --- | --- | --- | --- |
| **Study** | | **QuADS Score (/39)** | **Aims** | **Design & measures** | **Participants** | **Findings (by research question)** | | | |
|  |  |  |  |  |  | **Q1** | **Q2** | **Q3** | **Q4** |
| **Year of publication** | | **2011** | | | | | | | |
| Dawson, D.  (Scotland) | | 11 | To develop an accessible information strategy and produce ERHI within an *NHS Trust. | Service Improvement Project collating existing Trust ERHI, developing ERHI (dental) and ‘how to’ guide for producing Easy Read information; reported descriptively. | Development and review panel: people with Intellectual Disabilities, healthcare professionals (disciplines unspecified), dental staff, Carers and Corporate Communications (all numbers unspecified) | Developed ERHI using NHS Scotland Learning Disabilities Managed Care Network (2007)  guidance. | Resources were developed and reviewed by the panel.  Resources received considerable positive feedback. | Not applicable. | Not applicable. |
| Kelly, J.  (England) | | 14 | To report on experiences of implementing health education strategies to improve care for people with Intellectual Disabilities and diabetes. | Audit and Service Improvement Project developing Diabetes-related ERHI to be used during patient education groups, reported descriptively. | Resource developer: Nurses  Review panel: people with Diabetes and Intellectual Disabilities, Diabetes Specialist Nurses (all numbers unspecified) | Not reported. | Resources were reviewed by the panel.  Requests from other Trusts for resource pack to be shared. ERHI resource developed reportedly used as a model for other services. | Not applicable. | Anecdotal reports of increased patient understanding, reduced episodes of ill health, increased attendance at health checks and increased adherence with treatment plans. |
| King, S.  (England) | | 6 | To review policy for patients with Intellectual Disabilities to determine whether changes were required within the Surgical pre-assessment unit. | Service Improvement Project converting pre-admission assessment documents to Easy Read formats (including a case study) reported descriptively. | Resource Developer: Nurse  Review Panel: Intellectual Disability Nurse, Advocacy group (People with Intellectual Disabilities), Graphic Designer and Patient Information Group (all numbers unspecified) | Developed *ERHI using guidelines from Intellectual Disability Lead Nurse, Mencap (2002), and Department of Health (2003). | Resources were reviewed by the panel.  The review panel did not recommend any revisions. | Not applicable. | Not applicable. |
| **Year of publication** | | **2012** | | | | | | | |
| Howieson, J., Clarke, K.  (Scotland) | | 18 | To improve access to information about health screening and follow-up treatment for people with Intellectual Disabilities. | Service Improvement Project developing cancer related ERHI. Data collected using 3 focus groups and reported descriptively. | Resource developers: 2 Intellectual Disability Nurses  Working group: Nurses, Dietitians, Psychiatrists, Psychologists, Occupational Therapists, Physiotherapists, Speech and Language Therapy, Cancer Nurse Consultants, General Practitoners (all numbers unspecified)  Focus groups: ~30 members of a patient advocacy group. | Developed ERHI using Department of Health (2003) and Mencap (2008) guidelines. | The working group, and an external web agency, were consulted during the resource development process.  Patient focus groups advised on the layout, pictures, and language used in the ERHI and communication aid.  Resources received with considerable positive feedback. | Nurses developed a communication aid to be used alongside the ERHI: a hand-held picture book containing Makaton symbols. | Anecdotal reports of benefit in Intellectual Disability and non- Intellectual Disability cohorts including: hearing impairment, head and neck surgery, patients with English as a second language. |
| Porter, E., Kidd, G., Murray, N., Uytman, C., Spink, A., Anderson, B.  (Scotland) | | 17 | To develop accessible antenatal resources and to evaluate the acceptability and accessibility of the adapted resources from the perspective of women with ID and community midwives. | Service improvement project developing ERHI (maternity/ birthing) and  delivering training sessions covering Intellectual Disability and the use of ERHI.  Data gathered using structured interviews and evidence synthesised using thematic analysis. | Resource developers: Speech and Language Therapy service.  Resource evaluators: 5 women (mothers) with Intellectual Disabilities and their carers, 39 Midwives and Health Visitors. | Not reported. | The Speech and Language Therapy department consulted midwives during the development process.  Those using the ERHI felt that it had a clear format. | Midwives used ERHI as model to reduce sentence complexity.  Midwives supplemented ERHI with verbal explanation, but felt they needed additional guidance from Intellectual Disability Nurses to do this effectively. | Midwives reported that ERHI increased patient confidence and self-direction.  People with Intellectual Disabilities felt the adapted resources simplified information, increased understanding, and supported informed decision making. |
| **Year of publication** | | **2014** | | | | | | | |
| Chapman, H.  England | | 38 | To explore the effects of ERHI on the health consultation experience for people with Intellectual Disabilities. | Mixed methods qualitative study.  Data gathered using audio recorded observations, semi-structured interviews and focus groups.  Evidence synthesised using a Constructivist Grounded Theory approach. | 25 people with Intellectual Disabilities. | Not applicable. | Not applicable. | “Health Facilitators” wrote down key points and provided additional verbal explanation.  One practitioner effectively used physical demonstration. | Participants provided with ERHI reported having a sense of control over their health and were satisfied with the consultation.  Health Facilitators felt that ERHI was not sufficient without other forms of communication. |
| **Year of publication** | | **2015** | | | | | | | |
| Mander, C.  England | | 31 | To explore the current experiences of producing and delivering Accessible Information across a range of stakeholders | Qualitative scoping exercise. Data collected using 3 semi-structured focus groups and 4 semi-structured individual interviews.  Evidence synthesised using a framework approach to thematic analysis | 4 people with Intellectual Disabilities, 14 Healthcare Professionals: 7 Specialist Intellectual Disability Service staff, 4 mainstream public services staff, 3 Speech and Language Therapists. | 1 participant referred to “Government guidelines” on Easy Read. | There is a requirement for specialist expertise and / or an entire role dedicated to ERHI development.  ERHI needs to be adapted and personalised to suit to the individual, but this is not always feasible. | ERHI should not just be provided but “delivered” in a “dignified” style using “communication ramps”. | 1 participant reported that ERHI gives people with Intellectual Disabilities more confidence. |
| Marriott, A., Turner, S., Ashby, S., Rees, D.  England | | 9 | To describe the role of screening liaison nurses for adults with Intellectual Disabilities in relation to providing accessible information. | Case studies detailing the delivery of ERHI (bowel, breast and cervical screening), reported descriptively. | 3 screening liaison Nurses, 4 people with Intellectual Disabilities. | Not applicable. | Not applicable. | The screening liaison Nurse approach to delivering ERHI included: provision of accompanying verbal information, practical demonstration, and supplementing ERHI with additional images. | Anecdotal evidence that reasonable adjustments including ERHI may increase cancer screening uptake. |
| Rowlands, L.  Wales | | 27 | To assess whether individuals with an Intellectual Disabilities can read a sample Easy Read leaflet independently or if support is needed | Single case within-subject design using a pre-post questionnaire to evaluate the effectiveness of 2 ERHI resources (health checks), evidence synthesised qualitatively and quantitatively. | 6 people with Intellectual Disabilities, 1 nominated individual (support person). | Developed ERHI using Collis (2012) guidelines. | Simple Measure of Gobbledygook (SMOG) index calculated.  SMOG index of both samples between 13-14. | Nominated individual identified key terms and explained them. | For 5/6 participants. correct responses on a questionnaire increased following interaction with ERHI facilitated by a nominated individual. |
| **Year of publication** | | **2016** | | | | | | | |
| Denyer, M.  England | | 13 | To ensure equity of diabetes service provision across the local health community for people with diabetes and Intellectual Disabilities. | Service improvement project developing ERHI (Diabetes & related topics e.g. foot care) reported descriptively. | Resource developer: Specialist Intellectual Disability Nurse  Review panel: Community Intellectual Disability Nurse, People with Intellectual Disabilities, Intellectual Disability Healthcare Professionals (roles unspecified), community Intellectual Disability teams, Public Health colleagues, Speech and Language Therapists (all numbers unspecified). | Not reported. | The resource developers consulted the review panel when creating ERHI.  Reported national interest in resources. | Developed “Talking to me” prompt cards to encourage carers to simplify their vocabulary. | Not applicable. |
| Mander, C.  England | | 34 | To identify how nurses work with, and potentially adapt, accessible information and to determine the extent to which the delivery of accessible information influences understanding. | Non-participant observational study exploring the delivery of cancer related ERHI using Conversation Analysis, synthesised using cluster identification. . | 2 community Intellectual Disability Nurses, 2 women with Intellectual Disabilities. | Not applicable. | Not applicable. | Identified key interaction patterns and reported associated benefits. | ERHI alone did not appear adequate to support information needs.  Acquiescent responses, over-generalisation and misinterpretation noted.  1 participant did not demonstrate increased understanding with ERHI and became embarrassed and disengaged. |
| Yaneva, V., Temnikova, I., Ruslan M.  England | | 20 | To present an approach for automatic evaluation of the readability of text simplification output for readers with cognitive disabilities. | Pilot study of Easy Read Corpus, quantitative data gathered and reported. | N/A | Developed Easy Read information using Freyhoff et al., (1998) guidelines. | Used Flesch-Kincaid Grade Level (Kincaid et al., 1975) readability formulae.  19/353 documents (5%) discarded due to score >65. | Not applicable. | Not applicable. |
| **Year of publication** | | **2017** | | | | | | | |
| Sawhney, I., Adams, D., Zia, A.  England | | 18 | To determine if Intellectual Disability Psychiatrists routinely discuss, and give advice about, bone health, and the risks associated with antiepileptic medication, to patients/carers seen in epilepsy clinics. | Audit and Service Improvement Project developing ERHI (bones health), qualitative and quantitative data reported. | Audit of treatment of 50 people with Intellectual Disabilities.  Working group: Consultant Psychiatrist, Principal Pharmacist, Making Services Better advocacy group including people with Intellectual Disabilities, practice governance staff (all numbers unspecified) | Not reported. | The working group developed and reviewed information collaboratively.  Quality judgements not reported. | ERHI prompts clinicians to elaborate on key discussion points. | Not applicable. |
| **Year of publication** | | **2018** | | | | | | | |
| House, A., Latchford, G., Russell, A, M.; Bryant, L., Wright, J., Graham, E., Stansfield, A., Ajjan, R.  England | | 13 | To develop a supported self-management intervention for adults with a mild or moderate Intellectual Disability and type 2 diabetes. | Scoping exercise reviewing 18 ERHI resources using a structured questionnaire.  Pilot study employing own ERHI resource, data gathered using a structured questionnaire and synthesised qualitatively. | Review panel: Researchers, Intellectual Disability Dietitian, people with Intellectual Disabilities (all numbers unspecified). | Not reported. | Resources were reviewed by the panel and rated using a Likert scale.  The panel felt that the pilot resource was sufficiently easy to read. | 37% of people with Intellectual Disabilities had a “very engaged” main supporter to aid with interpretation of ERHI. | 30% of participants were deemed to be engaged by ERHI compared to 58% engaged by education sessions. |
| Wilson, J., Adeline, P., Bungaroo, D., Khadra, A., White, S., Bradley, E., Lodge, K-M., Hollins, S.  England | | 30 | To evaluate and compare the impact of two different educational interventions about testicular health on knowledge, skills and health-related behaviours. | Participatory randomised parallel study providing education on testicular examination via a teaching group and an EHRI group. Data collected using a Questionnaire (adptQ) and synthesised quantitively. | 39 men with Intellectual Disability: 20 in teaching group, 19 in leaflet group.  Steering group: men with Intellectual Disabilities, Speech and Language Therapy , psychology, Psychiatry, General Practice, community Intellectual Disability Nursing, urology specialist (all numbers unspecified) | Not reported. | The leaflet was designed collaboratively by members of the steering group and an artist with expertise in publishing materials in accessible formats.  Quality judgements not reported. | Discussion with family and Carers was encouraged. | Significantly improved knowledge and skills, self-efficacy, and confidence in ability to self-examine and seek help was gained after six months by those in the leaflet group. The intervention influenced associated health behaviours. |
| **Year of publication** | | **2019** | | | | | | | |
| Chinn, D.  England | | 36 | To examine how ERHI was employed by clinicians and received by patients. | Non‐consecutive case series, data gathered through video recordings/ transcription and synthesised using conversation analysis. | 32 GP primary care staff, 9 Specialist Intellectual Disability Nurses. | Not applicable. | Not applicable. | ERHI was used to introduce a topic and establish joint attention. ERHI acted as an aide‐memoire for staff. Provision of unsolicited advice was observed. | Not applicable. |
| Heslop, P., Turner, S., Read, S., Tucker, J., Seaton, S., Evans, B.  England | | 13 | To describe how three healthcare services have met the Equality Act (2010) duty to make reasonable adjustments for disabled people. | Service evaluation incorporating case studies detailing communication support provided to people with Intellectual Disabilities (ERHI for Abdominal Aortic Aneurysm screening and bowel cancer screening). | People with Intellectual Disabilities accessing care from 3 NHS Trusts (numbers unspecified). | Not reported. | Abdominal Aortic Aneurysm screening leads collaborated with the local Intellectual Disability service to develop ERHI.  Quality judgements not reported. | Intellectual Disability Nurses provided opportunities for additional verbal discussion.  Future plans included training for screening programme staff in basic Makaton. | 1 patient completed a bowel cancer screen successfully after engaging with ERHI. |
| **Year of publication** | | **2020** | | | | | | | |
| Buell, S., Langdon, P. E., Pounds, G., Bunning, K.  England | | 34 | To measure the effects of extrinsic and intrinsic factors on users’ comprehension of health information provided in adapted written “easy read” material. | Randomised Controlled Trial with four conditions comparing linguistically simple (easy read) or complex text with and without mediation  Data extracted using formal language assessment and synthesised quantitatively using descriptive statistics. | 60 people with Intellectual Disabilities.  Project advisory group (unspecified). | ERHI published by Department of Health used as templates. | Initial versions of the leaflet were reviewed by the project advisory group and suggestions implemented via group consensus. The resource was then reviewed by a professional familiar with ERHI.  Flesch-kincaid readability measures (Kincaid et al., 1975) were applied and iterative revisions made.  Revisions were required according to feedback from the project advisory group. | A script was used for mediation, based upon Palincsar & Brown’s (1984) model of reciprocal reading including 4 steps: summary, page by page clarification, questioning and predicative reasoning. | 8 questions were used to assess superficial recall, deep recall and inferential application of information.  Scores did not differ significantly between 1) mediation and non-mediation groups, or 2) linguistically complex and linguistically simple groups. |
| Chinn, D.  England | | 31 | To learn from ERHI creators about the practices they prioritise, the key content elements they consider, and their expectations for real-life use. | Individual, paired, and group interviews, evidence synthesised using thematic analysis. | 7 representatives from English third sector not-for-profit organisations, 1 service user consultant (person with Intellectual Disabilities), 1 nurse Academic, 1 NHS commissioner. | Developed ERHI using Change (2015) and Department of Health (2010) guidelines. | People with Intellectual Disabilities and medical professionals are employed as “quality checkers”.  There is often minimal input from people with Intellectual Disabilities, and limited opportunities for stakeholder feedback.  There was a great deal of variability in the ERHI. Resources emphasised compliance and excluded key medical information. | Highlighted the crucial role of the “literacy mediator” who provides technical help, interprets information, and initiates conversations. | Not applicable. |
| Kotwal, H., Fleming, J., Barlow-Stewart, K., Boyle, J., Silberbauer, L., Leffler, M., Murray, L., Palmer, EE.  Australia | | 28 | To evaluate the feasibility and acceptability of a booklet for people with Intellectual Disabilities to read with their carers prior to their genetics appointment. | Pilot evaluation of ERHI (genetics clinic booklet), qualitative data collected using a survey. | Resource developers: 6 genetic Counsellors, 1 Clinical Geneticist, 1 Project Coordinator  Review panel: 2 people with Intellectual Disabilities, 1 Easy Read ‘expert’. | Not reported. | Review panel provided recommendations for improved readability.  Readability of the booklet was assessed using readability software titled Readable.10.  Average readability score of 3.8 (judged accessible for people with mild Intellectual Disability).  The resource required 6 revision cycles before it was considered Easy Read.  83% of developers and reviewers felt the content was accurate, 76% felt that content was Easy Read, 86% would use the resource clinically. | Not applicable. | Not applicable. |
| Waight, M., Oldreive, W.  England | | 23 | To examine, with people with Intellectual Disabilities, different formats of accessible health information, and explore with them which formats they found useful. | Qualitative data gathered using 6 focus groups and synthesised using Grounded Theory. | 10 people with Intellectual Disabilities. | Referred to guidelines from the Norah Fry Research Centre and Royal National Institute of the Blind (2004), The Clear Communication People (2009), Department of Health (2010), International Federation of Library Associations and Institutions (2010), Belfast Health and Social Care Trust (2015), Change (2016). | Not applicable. | Nominated individuals use supportive strategies including explaining and repeating information. | Participants felt that images assisted in clarifying information. Participants highlighted the benefit of being able to return to written information. |
| **Year of publication** | | **2021** | | | | | | | |
| Cox, A., Parsons, T., Watkin, S., Gallagher, A.  England | | 17 | To describe the process of developing resources in collaboration with key stakeholders to support the workforce in delivering good maternity care to parents with Intellectual Disabilities. | Two phase mixed methods study developing and piloting ERHI (maternity/birthing), qualitative and quantitative data gathered using interviews and surveys. | Phase 1 (interviews):16 key stakeholders (4 midwives, 2 social workers, 2 support workers, 2 service managers, 2 Intellectual Disability Nurses, 2 parents with Intellectual Disabilities, Foster mother, Informal carer.  Phase 2 (pilot): 20 healthcare professionals: 8 midwives, Support manager, Student health visitor, Acute liaison Nurse, Consultant Obstetrician, Teaching fellow. | Not reported. | Research team and advisory group (research fellow, Nurse, 2 Teaching Fellows, Midwife, Team Manager, 2 Lecturers, Clinical Advisor, Social Worker, Clinical Psychologist, 3 people with Intellectual Disabilities, Carer, Expert by Experience, Agent for change, Producer, Programme manager) iteratively co-developed and reviewed resources.  Panel appraised ERHI:  Clear content (100%), Logical layout (100%), Appropriate for person with Intellectual Disabilities (73%). Amendments were suggested and actioned before finalising the resource. | Identified value in facilitators checking patient understanding of ERHI.  Highlighted benefit of practical and physical demonstrations.  Highlighted the value of having continued access to accessible information at home to review with family/carers. | Not applicable. |
| Doherty, P., Barksby, J., McCorkindale, M.  England | | 5 | To describe the process of developing a leaflet to inform people with Intellectual Disabilities about sepsis. | Service improvement project developing and implementing ERHI (sepsis), reported descriptively. | Resource developer (student Nurse), Review Panel: People with Intellectual Disabilities, family Carers, Healthcare Professionals – numbers unspecified) | ERHI developed using guidance from Mencap (2002), Marsay (2017) and Office for Disability Issues and Department for Work and Pensions (2018). | Review process not reported.  Review panel recommended revisions before finalising the resource. | Not applicable. | Not applicable. |
| **Year of publication** | | **2022** | | | | | | | |
| Dam, L. T., Heidler, P., King, I.  Austria | | 18 | To document the experiences of answering a pilot questionnaire to evaluate knowledge transfer. | Pilot study of a website containing ERHI (amongst other topics), qualitative and quantitative data collected using a structured questionnaire. | 10 people with mild-moderate Intellectual Disabilities. | Not applicable. | People with Intellectual Disabilities proofread the website content and tested its usability. The content was then adapted according to their feedback.  Review panel recommended revisions. | ‘Supporters’ present throughout to “familiarise” people with Intellectual Disabilities with the ERHI. | Most participants answered every health-related question correctly after engaging with the website. |
| Toussi, K., Y Cithambaram, K.  Ireland | | 17 | To evaluate the accessibility and readability of the written information provided to people with Intellectual Disabilities on COVID-19 and its vaccinations. | Content analysis of Covid-19 ERHI, reported descriptively. | Resource reviewers: Nursing student, Assistant Professor; School of Nursing (Authors). | Refers to recommendations on accessible information for people with Intellectual Disabilities by Paré and Kitsiou (2017), NHS England (2018) and Accessible Information Working Group (2011). | Quality appraised ERHI using self-developed tool based on NHS England (2018) and Accessible Information Working Group (2011) guidance according to parameters: general, font size, font type, and spacing. ERHI producers interpreted guidance differently and each document had varying strengths and limitations.  1 document met “nearly all” criteria and it was suggested this could be used as a template. | Not applicable. | Not applicable. |
| **Year of publication** | | **2023** | | | | | | | |
| Dam, L. T., Heidler, P., King, I.  Austria | | 24 | To improve knowledge transfer of health information for people with Intellectual Disabilities by creating a website prototype. | Feasibility study of a website containing ERHI (amongst other topics), qualitative and quantitative data collected using focus groups and unstructured interviews. | Resource developers: health Researchers (numbers unspecified)  Focus groups: 20 People with Intellectual Disabilities, 2 Carers | Refer to Inclusion Europe guidelines (n.d.) and Web Content Accessibility Guidelines. | People with Intellectual Disabilities provided feedback on the written and pictorial content focusing on comprehensibility. They performed quality assurance on printed versions.  Not feasible to fully comply with Easy Read guidance as this leads to more complex navigation in digital formats. | Not applicable. | Not applicable. |
| Douglass, E., Johnson, C., Lucas, G., Dowling, S.  England | | 26 | To gather the views of women with Intellectual Disabilities about infant feeding decision making and discuss the accessibility of currently available infant feeding resources. | Qualitative data on infant feeding ERHI gathered using a scoping review and focus group, data synthesised using reflexive thematic analysis and visual analysis. | 4 women with Intellectual Disabilities. | Not applicable. | Feedback regarding suboptimal image use and omissions of key medical information. | Not applicable. | Not applicable. |
| **Year of publication** | | **2024** | | | | | | | |
| Bruun, A., Cresswell, A., Jeffrey, D., Jordan, L., Keagan‐Bull, R., Giles, J., Swindells, S., Wilding, M., Payne, N., Gibson, S. L., Anderson‐Kittow, R., & Tuffrey‐Wijne, I.  England | | 24 | To co‐design a preliminary toolkit of end‐of‐life care planning  approaches and resources that can be implemented in adult social care services for people with Intellectual Disabilities. | Experience based co-design process, reported descriptively. | Co-design group: 9 people with Intellectual Disabilities, 5  family members, 5 Intellectual Disability support staff, 2 Intellectual Disability service  managers, 5 healthcare professionals, 3 organisational leads, 9 researchers. | Not applicable. | Co-design group tried and tested ERHI (end of life care).  The group did not engage with ERHI. They were overwhelmed with the number of pages, complicated vocabulary, and jargon. It was decided not to include ERHI in the toolkit, nor develop any new ERHI. | Not applicable. | Not applicable. |
| Buell, S., Pounds, G., Langdon, P., & Bunning, K.  England | | 28 | To explore the differences in language used between ERHI and the Standard Text versions of the same material. | Qualitative design employing Systemic Functional Linguistics, qualitative and quantitative findings reported. | Project advisory group (unspecified). | Not applicable | Used Flesch-Kincaid Grade Level (Kincaid et al., 1975) readability formulae and Coh-Metrix automated software.  Significantly reduced Grade Level score for ERHI compared to Standard Text, however readability scores were variable. Differences were observed between ERHI and standard text referential, personal, and textual linguistic functions. | Not applicable. | Not applicable. |
|  | Note:  * ERHI = Easy Read Health Information  * NHS = National Health Service | | | | | | | | |
