# Supplementary material for: Easy Read Health Information for People With Intellectual Disabilities: A Systematic Review of the Evidence
Source: J Appl Res Intellect Disabil. 2026 Feb 11;39(1):e70195. doi: 10.1111/jar.70195 (PMC12893875; doi:10.1111/jar.70195)
Supplement: Supplementary file 3 — Table S3: Stakeholders involved in the Easy Read Health Information development and review process. [file JAR-39-e70195-s004.pdf]

Supplementary file 3. *Stakeholders involved in the Easy Read Health Information development and review process.*

Stakeholders involved	Development process only	Review process only	Development and review process
Person(s) with Intellectual Disabilities	Dawson, 2011<br>Sawhney et al., 2017<br>Cox et al., 2021	King, 2011<br>Dawson, 2011<br>Kelly, 2011<br>Howieson & Clarke, 2012<br>Denyer, 2016<br>Sawhney et al., 2017<br>House et al., 2018<br>Kotwal et al., 2020<br>Cox et al., 2021<br>Doherty et al., 2021<br>Dam et al., 2022<br>Dam et al., 2023<br>Bruun et al., 2024	Dawson, 2011<br>Sawhney et al., 2017<br>Cox et al., 2021
Nurse(s)	King, 2011<br>Kelly, 2011<br>Howieson & Clarke, 2012<br>Porter et al., 2012<br>Denyer, 2016<br>Wilson et al., 2018<br>Cox et al., 2021<br>Doherty et al., 2021	King, 2011<br>Dawson, 2011<br>Kelly, 2011<br>Howieson & Clarke, 2012<br>Denyer, 2016<br>Cox et al., 2021	King, 2011<br>Kelly, 2011<br>Howieson & Clarke, 2012<br>Denyer, 2016<br>Cox et al., 2021
Doctor(s)	Howieson & Clarke, 2012<br>Sawhney et al., 2017<br>Wilson et al., 2018<br>Chinn, 2019<br>Cox et al., 2021	Howieson & Clarke, 2012<br>Sawhney et al., 2017<br>House et al., 2018*	Howieson & Clarke, 2012<br>Sawhney et al., 2017
Advocate(s)/advocacy group(s)	King, 2011<br>Howieson & Clarke, 2012<br>Sawhney et al., 2017	King, 2011<br>Howieson & Clarke, 2012<br>Sawhney et al., 2017	King, 2011<br>Howieson & Clarke, 2012<br>Sawhney et al., 2017
Carer(s)	Dawson, 2011<br>Cox et al., 2021<br>Dam et al., 2023	Dawson, 2011<br>Cox et al., 2021<br>Doherty et al., 2021<br>Bruun et al., 2024	Dawson, 2011<br>Cox et al., 2021
Researcher(s)	Cox et al., 2021<br>Dam et al., 2023	House et al., 2018<br>Buell et al., 2020<br>Cox et al., 2021<br>Dam et al., 2023<br>Bruun et al., 2024	Cox et al., 2021<br>Dam et al., 2023
Psychologist(s)	Howieson & Clarke, 2012<br>Wilson et al., 2018<br>Cox et al., 2021	Howieson & Clarke, 2012<br>Cox et al., 2021	Howieson & Clarke, 2012<br>Cox et al., 2021
Speech and Language Therapist(s)	Porter et al., 2012<br>Mander, 2015<br>Denyer, 2016<br>Wilson et al., 2018	Denyer, 2016	Denyer, 2016
“Health(care) professional(s)” unspecified	Dawson, 2011	Dawson, 2011<br>Denyer, 2016<br>Doherty et al., 2021<br>Bruun et al., 2024	Dawson, 2011

Corporate team(s)	Dawson, 2011<br>King, 2011	Dawson, 2011	Dawson, 2011
Higher Education Institution staff	Cox et al., 2021	Cox et al., 2021<br>Buell et al., 2020	Cox et al., 2021
Midwife/Midwives	Cox et al., 2021	Porter et al., 2012<br>Cox et al., 2021	Cox et al., 2021
Dietitian(s)		Howieson & Clarke, 2012<br>House et al., 2018	
Easy read Expert(s)		Buell et al., 2020<br>Kotwal et al., 2020	
Clinical Governance staff	Sawhney et al., 2017	Sawhney et al., 2017	Sawhney et al., 2017
Expert(s) by Experience	Cox et al., 2021	Cox et al., 2021<br>Bruun et al., 2024	Cox et al., 2021
External agency/agencies	Howieson & Clarke, 2012	House et al., 2018*	
Pharmacist(s)	Sawhney et al., 2017	Sawhney et al., 2017	Sawhney et al., 2017
Social Worker(s)	Cox et al., 2021	Cox et al., 2021	Cox et al., 2021
Service manager(s)	Cox et al., 2021	Cox et al., 2021<br>House et al., 2018<br>Bruun et al., 2024	Cox et al., 2021
Artist(s)	Wilson et al., 2018	House et al., 2018	
Graphic Designer(s)		King, 2011	
Occupational Therapist(s)		Howieson & Clarke, 2012	
Patient information group(s)		King, 2011	
Physiotherapist(s)		Howieson & Clarke, 2012	
Public Health Organisation(s)		*Denyer, 2016*	
